# Multiomics analysis reveals the genetic and metabolic characteristics associated with the low prevalence of dental caries

**DOI:** 10.1080/20002297.2023.2277271

**Published:** 2023-11-02

**Authors:** Jinshen Liu, Si-Ying Ye, Xin-Dong Xu, Qiulin Liu, Fei Ma, Xueting Yu, Yu-Hong Luo, Ling-Ling Chen, Xiaojuan Zeng

**Affiliations:** a College of Stomatology, Hospital of Stomatology, Guangxi Medical University, Nanning, China; b State Key Laboratory for Conservation and Utilization of Subtropical Agro-bioresources, College of Life Science and Technology, Guangxi University, Nanning, China

**Keywords:** Baiku Yao, dental caries, metabolome, metagenomic, multiomics, antimicrobials

## Abstract

**Background:**

Despite poor oral hygiene, the Baiku Yao (BKY) ethnic group in China presents a low prevalence of dental caries, which may be related to genetic susceptibility. Due to strict intra-ethnic marriage rule, this ethnic has an advantage in studying the interaction between genetic factors and other regulatory factors related to dental caries.

**Methods:**

Peripheral blood from a caries-free adult male was used for whole genome sequencing, and the BKY assembled genome was compared to the Han Chinese genome. Oral saliva samples were collected from 51 subjects for metabolomic and metagenomic analysis. Multiomics data were integrated for combined analysis using bioinformatics approaches.

**Results:**

Comparative genomic analysis revealed the presence of structural variations in several genes associated with dental caries. Metabolomic and metagenomic sequencing demonstrated the caries-free group had significantly higher concentration of antimicrobials and higher abundance of core oral health-related microbiota. The functional analysis indicated that cationic antimicrobial peptide resistance and the lipopolysaccharide biosynthesis pathway were enriched in the caries-free group.

**Conclusions:**

Our study provided new insights into the specific regulatory mechanisms that contribute to the low prevalence of dental caries in the specific population and may provide new evidence for the genetic diagnosis and control of dental caries.

## Introduction

It is estimated that 2.5 billion adults and 573 million children worldwide suffer from untreated dental caries, which imposes a considerable health burden on healthcare systems and on society at large [1]. Dental caries is a chronic disease influenced by multiple factors, mainly triggered by ecological imbalances microbes within the biofilm covering the tooth surface, starting with complex microbial interactions within the biofilm and being influenced by food, host genetics, and time [2,3]. The pathogenesis of dental caries is very similar across patients, and *Streptococci mutans* and *Lactobacillus acidophilus* are considered the main microbes associated with dental caries [4]. However, *S. mutans* and *L. acidophilus* are not universally present in all dental caries patients and are only a part of the complex microbial community [5]. Studies have demonstrated that the onset of dental caries may be associated with *Veillonella*, *Actinomyces*, and *Prevotella* [6–8]. These bacteria produce weak organic acids as metabolic byproducts of fermentable carbohydrates within dental biofilms, which can cause the localized pH to decrease below a critical value, resulting in dental demineralization and dental caries [9].

Genetic factors have been suggested to account for approximately 30–50% of the progression of dental caries [10]. The Baiku Yao (BKY) ethnic group, one of Chinese ethnic groups that transitioned from primitive to modern society, has a distinct cultural heritage and has been recognized by UNESCO for its preservation of ethnic traditions. The BKY population in Guangxi province has a strict intra-ethnic marriage rule and unique living customs, with a low genetic diversity of population. More importantly, the dental caries prevalence of BKY population is lower than the national average and the prevalence of the Chinese Han residents with similar living habits and dietary pattern (the main staple food is grain) in the same area [11]. It’s found that the frequency of sweet intake ≥ 2 times/week was 53.1% in the BKY, which was lower than that of Han (75.8%), and the results of the analysis of the association between environmental factors and caries showed that the factors influencing caries in this population in this region were mainly gender, sugar intake rate, and dental calculus [12]. Meanwhile, the association analysis between gene polymorphisms and caries suggested that polymorphisms in genes such as *DEFB1* were associated with a high risk of caries [13]. Nevertheless, there is currently a lack of available genomic data on this specific ethnic population for comprehensive characterization of genetic differences. Advancements in sequencing technology and genome assembly algorithms have led to significant enhancements in the completeness and quality of human genome assemblies in recent years [14]. But here is a significant shortage of high-quality reference genomic data with population specificity. Constructing a population-specific reference genome can enhance the accuracy and sensitivity of genetic variant detection, thereby providing more accurate assistance for genetic diagnosis [15]. Thus, it is essential to construct a high-quality, population-specific reference genome. An increasing number of studies are currently devoted to addressing this gap [15–17].

Previous studies have suggested that certain oral microbes in caries-free adults exhibit antagonistic effects on caries-causing microbes, providing protection against pathogens and inhibiting the onset and progression of dental caries [18,19]. Salivary antimicrobial substances also impede the growth of caries-causing microbes [20]. However, it remains unclear whether the BKY population harbors caries-inhibiting microbes, and the molecular mechanisms underlying their impact on caries onset in saliva and biofilm environments are unknown. Metagenomic sequencing enables comprehensive assessment of microbial community composition and facilitates the identification of functional genes and their potential roles in diverse environments [21]. Metagenomic sequencing has revealed highly diverse oral microcosms [18]. Analysis of the saliva metabolome can diagnose dysregulation-related diseases and reflect the molecular phenotype of oral biofilms in real time [22]. However, little research has been conducted to identify and characterize unique oral metabolites in carious and healthy populations, which play a crucial role in maintaining homeostasis between oral microbes and human hosts [23]. Liquid chromatography-tandem mass spectrometry (LC- MS/MS) metabolomic analysis of population saliva samples allows the exploration of key salivary metabolites that inhibit the growth of caries-causing colonizing microbes [24]. MS-based metabolomics offers highly sensitive quantitative analysis, detecting low-molecular-weight compounds at concentrations below the ng/mL range [25]. Additionally, LC-MS enhances the separation of complex mixtures, optimizing the detection of individual compounds [26].

This study aims to explore the genetic variations related to dental caries between BKY and Han ethnic groups and the regulatory mechanisms and influences of genetic variations, microbes, and metabolites on the occurrence of dental caries.

## Materials and Methods

### Study subjects

A survey of oral health was conducted on BKY adolescents at Li Hu Middle School in Nandan County, China. Peripheral blood from one caries-free adult male from the same region and ethnic group was collected for whole-genome sequencing. 27 caries-free subjects and 24 subjects with dental caries were further randomly selected to provide saliva samples for metabolome sequencing. Additionally, six samples were randomly selected from each of the caries-free and dental caries groups for metagenome sequencing. Subjects (1) were 12–15-year-old children of the BKY ethnic group, with three generations of ancestors (grandfather, grandmother, maternal grandfather, maternal grandmother, father, mother, and the child themselves) belonging to the BKY ethnic group (ethnicity determined based on household registration information), with an age range of ≥12 years and ≤15 years at the time of the survey; (2) were permanent residents in the local area without a history of residing elsewhere for more than 6 months; (3) had early permanent dentition: presence of all permanent teeth (possible presence of second molars; absence of third molars). Subjects were excluded if they (1) wore orthodontic appliances or attachments; (2) had used antibiotics, immunosuppressants, fluoride, or microecological regulators within 6 months; (3) had systemic diseases requiring long-term medication, such as diabetes, cancer, or cardiovascular disease; (4) had history of oral medication or surgical treatment within the last 6 months.

### Sample collection and oral examination

Referring to the Human Microbiome Project [27], participants were not allowed to eat, smoke, or drink alcohol 30 min prior to waking up in the morning for sampling. The dentist opened the sterile saliva collection tube and collected 2 ml non-stimulated saliva. Then, the tube was marked and stored in the − 80°C refrigerator immediately. The dentist performed the examination with reference to the diagnostic criteria and examination methods of the WHO guidelines [28] and recorded the number of decayed, missing, and filled teeth (DMFT). The poll followed the principles articulated in the Helsinki Declaration. The Guangxi Medical University College of Stomatology Ethical Review Committee approved the study protocol. All subjects provided written informed consent.

### Blood DNA extraction, library construction and sequencing

The blood sample used in the study were obtained from a healthy adult male participant belonging to the BKY ethnic group. The SQK-LSK109 kit from Oxford Nanopore Technologies was utilized for library construction to generate DNA libraries following the manufacturer’s standard protocol (Nanopore). The Oxford Nanopore PromethION sequencer was used to sequence the libraries, resulting in a total of 151,728,810,548 bp of raw sequencing data. The generated sequencing data was subjected to filtration based on sequence filtering criteria, with sequences exhibiting mean mass ≤ 7 being excluded, resulting in 142 Gb of data for BKY genome assembly. MGI Easy was utilized for library construction to generate DNA libraries following the manufacturer’s standard protocol (Illumina), and the high-throughput sequencing platform DNBSEQ was used to sequence the libraries, generating 150-bp pair-end reads totaling 203,476,703,400 bp.

### BKY genome assembly

The first step in this study was to use Flye (v2.9.1-b1780) to produce a preliminary assembly of contig-level chromosomes from ONT reads [29]. Subsequently, Pilon (v1.24) was employed to polish the contigs derived from the initial assembly using Illumina reads [30]. Finally, the chromosome scaffolder module (v4.0.5) in MaSuRCA was utilized to scaffold the contigs that had been polished after the postassembly process to the chromosome level, using the Han1 genome as a reference [31]. Quast (v5.2.0) [32] and Merqury (v1.3) [33] were used to mount the postScaffolds and perform a quality evaluation.

### Annotation of BKY genome

To map the gene structure annotations of the CHM13 reference genome to the newly assembled BKY genome in this study, Liftoff(v1.6.2) was employed [34]. EDTA(v2.1.2) was utilized to enhance the annotation of the BKY reference genome by incorporating repetitive sequences at the chromosome level [35]. To evaluate the accuracy of the gene annotations, LiftoffTools(v0.4.3) [36] and Mcscan (Python version) [37] were utilized to analyze the co-linearity of gene sequences with protein sequences between the BKY and CHM13 genomes, respectively.

### Comparative analysis of BKY and Han1 genome

MUMmer(v4.0.0rc1) was utilized to perform a comparative analysis of the BKY and Han1 genomes [38]. Specifically, the nucmer tools in MUMmer were used to align the genomic sequences of BKY and Han1. The SyRI(v1.6.3) tool was employed to detect resultant variants from the nucmer alignment results [39]. Plotsr was used to visualize BKY covariance with the Han1 genome [40]. Mum & Co(v3.8) was used to count and classify the number of genome-wide structural variants [41]. Mauve(v2015-02-26) was used for gene sequence alignment [42], Mauve and genoPlotR were used to visualize the covariance analysis of genes [43]. MAFFT [44] and Clustal Omega [45] were used for multiple sequence alignment of gene sequences and protein sequences, respectively. GeneDoc was used to present the results of multiple sequence alignment [46], and Integrative Genomics Viewer (IGV) was used to view the coverage of genomic region reads [47].

### In silico protein prediction

In this study, AlphaFold2 (v2.2.0) was utilized to model the 3D structure of candidate proteins from amino acid sequences [48]. Chimera (v1.17) was used to model the Protein Data Bank (PDB) format files of the resulting proteins [49]. Protscale (https://web.expasy.org/protscale/) was employed for protein hydrophobicity prediction.

### Sample preparation for metabolome

To remove the protein, the saliva samples were thawed at 4°C and 100 L aliquots were mixed with 400 L of cold methanol/acetonitrile (1:1, v/v). The mixture was centrifuged for 20 minutes (4°C 14,000 g). A vacuum centrifuge was used to dry the supernatant. The samples were re-dissolved in 100 L of acetonitrile/water (1:1, v/v) solvent and centrifuged at 14,000 g at 4°C for 15 minutes before being injected with the supernatant.

### LC‒MS/MS analysis

The samples were separated using a UHPLC (1290 Infinity LC, Agilent Technologies) HILIC column and analyzed by electrospray ionization (ESI) quadrupole time-of-flight mass spectrometry (AB Sciex TripleTOF 6600) in both positive and negative ionization modes. ProteoWizard software was used to convert the raw data to mzXML format, and XCMS software was used to conduct peak alignment, retention time correction, and peak area extraction. On the XCMS-extracted data, metabolite identification and data preparation were performed, followed by experimental data quality evaluation and data analysis. The metabolites in the biological samples were structurally identified by comparing the retention times, molecular masses (within 25 ppm molecular mass error), secondary fragmentation spectra, and collision energy of the metabolites in a local self-built standard database (in-house database (Shanghai Applied Protein Technology)) [50].

### Metagenomic sequencing and data filtering

The MGI Easy Universal DNA Library Prep Set User Manual was used to prepare high-quality DNA for sequencing libraries. On the DNBSEQ T7 platform, whole-genome shotgun sequencing was performed, yielding PE150 reads. All raw data were trimmed with SOAPnuke (v.1.5.2) [51] to remove reads with more than 10% N bases, adapter sequences, and reads with more than 50% low-quality bases (Q < 20). Following quality control, the remaining reads were aligned to the host genome with the Bowtie2 program [52], and reads that matched the human genome were deleted before further analysis of high-quality data.

### Extraction of microbiome DNA

Transfer 100–200 mg sample to the centrifuge tube with grinding beads. Add 1 mL Buffer ATL/PVP-10, grind the sample with the grinding machine, and incubate at 65°C for 20 min. The centrifuged samples were subjected to DNA extraction. A DNA BR kit with an enzyme-labeled test and agarose gel electrophoresis were used to assess DNA quality. Prior to further analysis, DNA samples were resuspended in TE buffer and kept at −80°C.

### Assembly and annotation of metagenomic data

MEGAHIT was used to construct high-quality reads from scratch [53]. Assembled contigs with lengths less than 200 bp were eliminated (parameters: -min-count 2 –k-min 93 –k-max 133 –k-step 10 –no-mercy – min-contig-len 200). MetaGeneMark was used to predict genes over contigs [54]. CD-HIT [55] was used to eliminate redundant genes, using identity and coverage cutoffs of 95% and 90%, respectively (parameters: -aS 0.9 -c 0.95 -d 0 -g 1). The abundance levels of several genes were measured using the transcripts per million (TPM) value by Salmon [56]. The BLASTp function of Diamond [57] was used to perform functional annotation of nonredundant genes using the KEGG, COG, and SwissProt databases.

### Species annotation and differentially abundant microbe identification

Clean data reads were mapped to the NCBI NT database, and species annotation was conducted using Kraken2 [58] with the default parameters. To generate taxonomic and functional abundance profiles, Bracken [59] software was used with the default settings. The Chao1 index was calculated by the ‘vegan’ package in R software to characterize α-diversity. PLS-DA based on Bray‒Curtis distance was used to measure variations in diversity between samples. To screen for significant variations in microbes and functions between groups (*P* value < 0.05), Wilcoxon’s rank sum test for the abundance and functional relative abundance of all species was performed using R software.

### Multivariate statistical analysis for metabolomics

To observe the overall distribution trends and differences between groups, the samples were subjected to principal component analysis (PCA) analysis using R. For supervised group comparison, 7-fold cross-validation was utilized to develop an evaluation parameter (Q2 > 0.5) using orthogonal partial least-squares discriminant analysis (OPLS-DA). Permutation tests were performed on the OPLS-DA models to avoid overfitting during model development. Each variable’s variable significance in the projection (VIP) value in the OPLS-DA model was calculated to indicate its contribution to categorization. As significant differential metabolites, metabolites with VIP > 1 and *P* value < 0.05 were chosen. The Fisher’s Exact Test was used to determine the level of significance of differential metabolite enrichment in specific KEGG pathways. The link between significant differential metabolites and differential microorganisms was investigated using Spearman’s correlation analysis.

## Results

### The completion of the first BKY genome expands existing human genome resources

**Figure 1.**
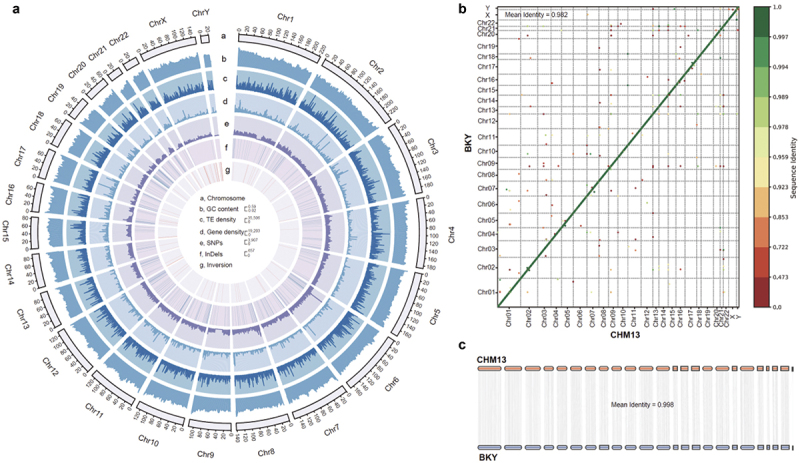
Overview of the *Homo sapiens* ‘BKY’ genome. a: circos plot of the *H. sapiens* ‘BKY’ genome. a. Chromosomes; b. GC content; c. TE density; d. gene density; e. SNPs; f. InDels; g. inversion. b: gene order plot of BKY and CHM13 gene sequences. The color of the point corresponds to the sequence identity between the reference gene and the target gene, where green indicates higher identity, and red indicates lower identity. c: synteny between BKY and CHM13 based on protein sequences.

In this study, we selected one healthy BKY’s adult male with caries-free for genome sequencing. In addition, we conducted metabolome sequencing in saliva samples from 51 subjects, including 24 from the caries group and 27 from the caries-free group, with an age distribution of 12–15 years (Supplementary Table S1). There were no significantly difference in age and sex between two groups (*P* value > 0.05). Additionally, we randomly selected 6 samples from the two groups for metagenome sequencing. A total of 142.78 Gbp (~49.75× coverage) of whole-genome sequencing data was obtained from the Oxford Nanopore Technology (ONT) platform and 203.48 Gbp (~70.89× coverage) of whole-genome sequencing data was obtained from the Illumina platform for the BKY genome. ONT reads were utilized to obtain a preliminary genome assembly at the contig level, which was further refined through polishing using Illumina sequencing data. The contigs obtained after polishing were then scaffolded using the T2T genome of Southern Han Chinese (Han1) as a reference, resulting in 99.43% (2.85 Gbp) of the contig bases being scaffolded onto 24 chromosomes, including autosomes (1–22) and sex chromosomes (X and Y) (Figure 1a). Furthermore, a low sequencing depth, with ~ 26.65× coverage, resulted in the Y chromosome being assembled to a size of 21 Mbp. Consequently, the final size of the assembled BKY genome was 2.87 Gbp. The contig N50 and scaffold N50 values were 28.86 Mbp and 151.1 Mbp, respectively, indicating high genome continuity (Supplementary Table S2). The results presented above confirm that the BKY genome assembly produced in this study is characterized by both high accuracy and excellent continuity.

Based on the repeat annotation results, 35.48% of the sequences in the BKY genome were identified as repeats. Subsequently, Liftoff was employed to transfer the annotation from the reference genome CHM13 to the BKY genome. As a result, a total of 54,086 genes were annotated, encompassing 19,583 protein-coding genes and 15,179 long noncoding RNA (lncRNA)-coding sequences. This annotation level is comparable to that of the current human genome, serving as further evidence supporting the thoroughness and precision of the BKY genome assembly (Supplementary Table S3).

The collinearity analysis of gene sequences obtained from the annotation of the BKY genome with CHM13 gene sequences [14] showed an average identity of 98.2% (Figure 1b). Subsequently, a comparative analysis was conducted on the protein sequences translated from the protein-coding genes in both the BKY and CHM13 genomes. The results of this analysis revealed a high degree of similarity between the protein sequences annotated in the BKY genome and their corresponding protein sequences in the CHM13 genome, with an average identity of 99.8% (Figure 1c). The results of functional annotation analysis indicated that 96.5% of the protein sequences could be accurately annotated using either homologous sequences or protein domains. In summary, this study produced a high-quality BKY genome assembly and corresponding annotations, which can be used for further genomics studies.

### Comparative genomic analysis reveals genetic variations in caries-related genes

**Figure 2.**
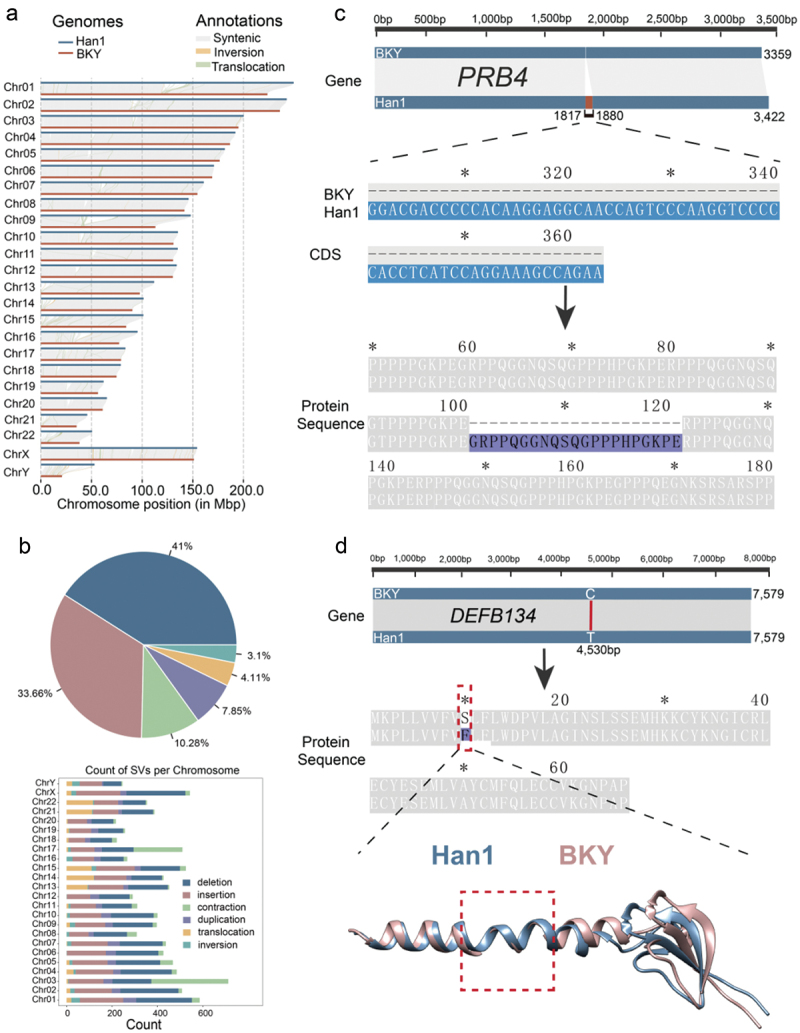
Comparison and variant calling in the BKY and Han1 genomes. a: collinearity of the Han1 genome with the BKY genome. b: classification and statistics of structural variants in the whole BKY genome and on each chromosome. c: comparison of *PRB4* between the BKY and Han1 genomes. From top to bottom: gene, CDS and protein sequence. The covariance region is shown in dark blue, and the variance region is shown in red. d: comparison of *DEFB134* between the BKY and Han1 genomes. From top to bottom: gene, protein sequence and protein structure, the calculated RMSD was 0.988 Å, and TM-score was 0.694.

To date, the sequencing of the publicly available Southern Han population genome (Han1) has reached the T2T level, with high assembly quality [16]. To determine the genetic basis of the variation in the onset of dental caries between the BKY and Han populations, we performed a comparative analysis of the linear genome structures of both groups. Collinearity analysis conducted between the Han1 and BKY genomes revealed a high degree of collinearity among all chromosomal regions except for the highly repetitive centromeric region, which showed partial loss of covariance between the two genomes (Figure 2a). A total of 9,390 structural variations (SVs) were identified, consisting of 3,850 deletions (41%), 3,161 insertions (33.66%), 965 contractions (10.28%), 737 duplications (7.85%), 386 translocations (4.11%), and 291 inversions (3.1%) (Figure 2b and Supplementary Table S4).

Further analysis was conducted on 70 caries-related genes, which included genes encoding proline-rich proteins and defensin (DEF) family genes encoding antimicrobial peptides in the BKY and Han1 genomes. The analysis reveals variations in the coding sequences (CDS) of 17 candidate genes. Further comparative analysis was conducted on the protein sequences encoded by the 17 genes with CDS variants, and variants were identified in a total of 7 protein sequences, including *PRB4* and *DEFB134* (Supplementary Table S5). The reliability of these detected variations was confirmed by examining the reads coverage in the gene regions in the BKY genome (Supplementary Fig. S1-4). The gene *PRB4* encodes a salivary protein rich in proline, in this study, a 63-bp deletion was observed in the CDS of this gene in BKY compared to the sequence in Han1, resulting in the loss of 21 residues (Gly111-Glu121) in the protein encoded by this gene in BKY compared to Han1 (Figure 2c). Additionally, *PRB1* and *PRB3*, which also code for proline-rich proteins, show deletions in BKY in comparison to Han1, resulting in smaller peptides within the encoded proteins (Supplementary Fig. S5A-B). The *DEFB134*, which encodes a defensin, also drew our attention. A single nucleotide mutation was observed in the coding region of this gene, where a T was converted to a C compared to Han1. This alteration caused a substitution of Phe10 with Ser10 in the protein sequence encoded by this gene, resulting in the replacement of a nonpolar amino acid with a polar one (Figure 2d). Additionally, the *DEFB126*, also known for encoding a defensin, exhibits a single nucleotide mutation in its coding sequence. As a result, the 71st threonine was converted to methionine in the protein sequence of DEFB126 in BKY, causing a polar change (Supplementary Fig. S5D). *ZNF160*, exhibits a single nucleotide mutation in its coding sequence. As a result, the 82st proline was converted to arginine in the protein sequence of ZNF160 in BKY (Supplementary Fig. S5E). Furthermore, *ZSWIM6*, associated with dental caries formation, was found to have a deletion of 6 bp in its CDS in BKY compared to Han1, leading to the loss of two glycines in its protein sequence (Supplementary Fig. S5C).

To further elucidate the impact of the variants in the candidate genes, protein structures with protein sequence variants between the BKY and Han1 genomes were predicted and compared in the study. The results showed that there are differences in the protein structures of the DEFB134, DEFB126, ZNF160 (Figure 2d and Supplementary Fig. S5C-D). Among them, DEFB134 was found to show significant structural differences between the BKY and Han1 populations (Figure 2d).

### Differences in antimicrobial metabolite abundance in the oral cavity revealed by metabolomic analysis

**Figure 3.**
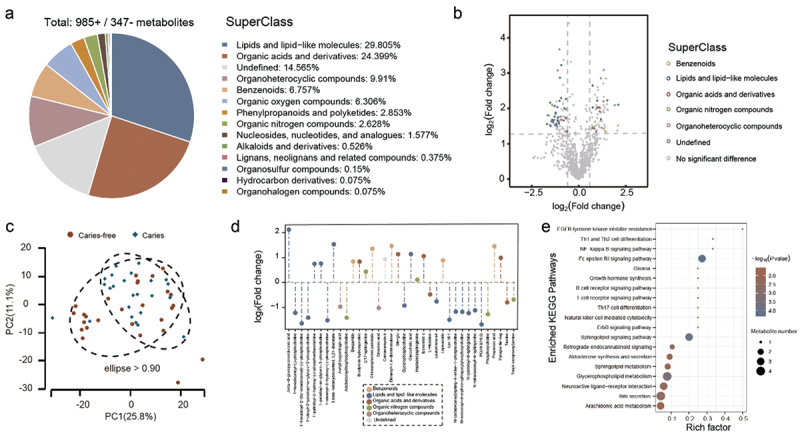
Comparison of metabolite profiles between the caries and caries-free groups in the BKY population. a: classification of identified metabolites. n = 1,332. b: volcano plot of saliva sample comparison (caries/caries-free). Significantly differentially expressed metabolites: FC > 1.5 or FC < 0.67; *p* value < 0.05. c: PCA of the caries and caries-free groups. n = 51. d: Fold changes and classification of significantly differentially expressed metabolites between the caries and caries-free groups. e: KEGG enrichment plots of the differentially expressed metabolites.

We performed a metabolomic analysis of saliva samples from 51 subjects using LC‒MS/MS. Our analysis involved both positive ion mode (ES+) and negative ion mode (ES-) LC/MS for the detection of metabolites. From these analyses, we detected 19,337 metabolites in total, 1,332 of which were mapped to known chemical structures (Supplementary Table S6). Among the annotated metabolites, lipids and lipid-like molecules (29.805%) and organic acids and their derivatives (24.399%) were identified as predominant (Figure 3a). Using univariate analysis, we analyzed all the annotated metabolites associated with differences between the groups. We identified 83 significant differentially expressed metabolites (fold change > 1.5 or < 0.67, *p* value < 0.05) between groups, while the majority of metabolites showed no significant difference (Figure 3b and Supplementary Fig. 6). Notably, the differentially expressed oral metabolites between groups with and without dental caries in the BKY population included primarily lipids and lipid-like molecules, organic acids and their derivatives, benzenes, and other substances (Figure 3b and Supplementary Fig. 6). Additionally, principal component analysis (PCA) revealed clear visual separation between the caries and caries-free groups (Figure 3c and Supplementary Fig. 7A). To further differentiate metabolites between groups, we conducted orthogonal partial least-squares discriminant analysis (OPLS-DA) of the data (Supplementary Fig. 7B-C). The validation of the model did not show any overfitting issues, indicating that it could describe the sample well and be applied to further data analysis (Supplementary Fig. 8). Further screening for significantly differentially expressed metabolites between the caries and caries-free groups was conducted using a combination of the variable importance of the projection (VIP) > 1 and *p* values < 0.05 based on the peak area determined by the OPLS-DA model. A total of 107 significantly differentially expressed metabolites were identified from saliva samples (Supplementary Table S7), including phosphatidylcholines (PCs) such as PC (16:0/16:0) and 1-hexadecyl-2-(9z-octadecenoyl)-sn-glycero-3-phosphocholine, lysophosphatidylcholines (LPCs) such as 1-stearoyl-2-hydroxy-sn-glycero-3-phosphocholine and LPC 18:1, and sphingolipids such as N-nervonoyl-d-erythro-sphingosylphosphorylcholine and *N*-(octadecanoyl) sphing-4-enine-1-phosphocholine. The abundance of these metabolites was significantly higher in the caries group (Figure 3d). The levels of small-molecule peptides such as Gln-Glu and Pyroglu-Ile-Arg were found to be higher in the caries-free group than in the caries group. Moreover, metabolites such as concanamycin b and benzamides were also observed to be more abundant in the oral saliva of the caries-free group in the BKY population (Figure 3d). The significantly affected metabolic and signaling pathways were identified by KEGG pathway enrichment analysis. Differentially expressed metabolites were mainly enriched in the glycerophospholipid metabolism, Fc epsilon RI signaling, sphingolipid signaling, sphingolipid metabolism, and arachidonic acid metabolism pathways (Figure 3e).

### Composition and function of the oral microbiota identified by metagenome sequencing

**Figure 4.**
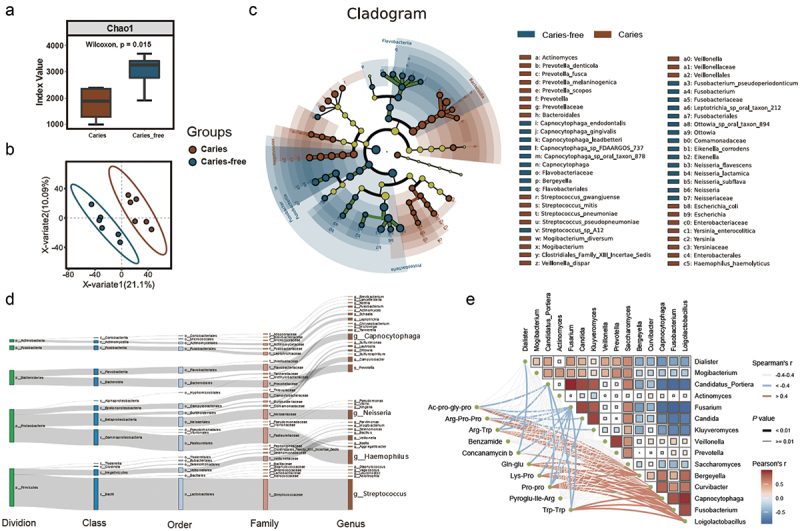
The composition of the oral microbiota is related to the onset of dental caries and metabolite abundance. a: PLS-DA clustering for oral saliva samples from 12 persons, where each point represents a single individual. b: richness (Chao1) indexes of the caries and caries-free groups. The *P* values were determined by the Wilcoxon test. c: differentially abundant microbes between the caries and caries-free groups identified by LEfSe analysis. d: taxonomic origin of the CAMP resistance gene cluster. e: correlation analysis between the abundance of antimicrobial metabolites and microbes. Microbial abundance was calculated at the genus level.

We conducted metagenomic sequencing and assembly based on 12 saliva samples from caries-free and caries individuals of the BKY ethnic group. The contig N50 for the assembled fragments ranged between 1,325 and 1,948 bp. The de novo prediction of coding genes in the contigs and the removal of redundancy were performed using MetaGeneMark, yielding a total of 622,381 nonredundant genes. Additionally, species annotation and abundance calculations were carried out by comparing raw reads from clean data with the NCBI NT database. Remarkably, rich diversity of bacterial species was detected in the oral saliva samples, comprising a total of 47 phyla, 93 orders, 201 families, 424 genera, and 4713 species/strains (Supplementary Fig. 9A). The Chao1 index was used to measure the species abundance of oral microbes in both the caries-free and caries groups of the BKY population (Figure 4a). The results showed that the diversity of the oral microbiota was higher in the caries-free group than in the caries group (*P* value = 0.015). Partial least squares discriminant analysis (PLS-DA) was performed at the phylum, genus, and species levels for both caries-free and caries groups of the BKY population with great differences in all levels (Figure 4b and Supplementary Fig. 10). The microbial communities with differential distributions between the caries and caries-free groups in the BKY population were statistically and visually analyzed using LEfSe analysis (Figure 4c and Supplementary Fig. 9B). At the genus level, the caries group exhibited high levels of *Prevotella*, *Veillonella*, *Actinomyces*, and *Mogibacterium*, whereas the caries-free group was enriched with *Neisseria*, *Fusobacterium*, *Capnocytophaga*, and others (Supplementary Table S8). At the species level, the caries group showed enrichment of *Prevotella melaninogenica*, *Streptococcus mitis*, *Veillonella dispar*, *Prevotella scopos*, and *Streptococcus pneumoniae*. On the other hand, *Neisseria subflava*, *Fusobacterium pseudoperiodonticum*, *Neisseria flavescens*, *Capnocytophaga endodontalis*, and *Capnocytophaga gingivalis* were more abundant in the caries-free group (Supplementary Table S9).

By functional annotation of microbial genes using databases such as KEGG, COG, and SwissProt, 53.1% of the genes were annotated with functional information for known homologous genes. We used the intergroup differential analysis software STAMP to analyze the differences in microbial functions between samples (Supplementary Fig. 11). Compared with the caries group, the caries-free group had a greater number of genes associated with cationic antimicrobial peptide (CAMP) resistance, lipopolysaccharide biosynthesis, biotin metabolism, the phosphatidylinositol signaling system, sulfur metabolism, D-amino acid metabolism, tryptophan metabolism, and lysine degradation. Further analyses revealed that the CAMP resistance gene cluster is primarily derived from *Streptococcus*, *Haemophilus*, *Neisseria, and other genera* (Figure 4d). *Streptococcus mutans*, *Lactobacillus acidophilus*, and *Actinomyces viscosus*, almost completely lack this gene cluster (Supplementary Table S10).

We performed a Spearman correlation analysis of metabolites and microbes that were significantly different between the caries-free and caries groups of the BKY population (Figure 4e). Most of the significantly differentially expressed metabolites were negatively correlated with reported caries-associated microbes (*Candida*, *Dialister*, *Mogibacterium*), including concanamycin b, benzamides, and most small-molecule peptides, such as Ac-Pro-Gly-Pro, Arg-Pro-Pro, Gln-Glu, Lys-Pro, and Pyroglu-Ile-Arg. Conversely, this class of compounds was positively correlated with the abundance of microbes including *Loigolactobacillus* and *Capnocytophaga*.

## Discussion

Dental caries is a chronic disease that adversely affects oral health in humans. The existing literature indicates that its onset is influenced by multiple factors, including genetics, dietary habits, and living environment [3]. Genetically, genes such as *PRB4* [60] and *DEFB1* [61] have been reported to be associated with the progression of dental caries due to variation. Therefore, understanding the genetic basis for the differences in its onset among different populations can contribute to the development of new prevention and treatment strategies for dental caries. Based on the results of a previous survey, it was discovered that the incidence of dental caries in the BKY population was significantly lower than that in the Han population under similar living conditions [11]. To elucidate the genetic basis of the difference in the onset of dental caries between the two groups, the first high-quality genome assembly of the BKY population was completed in this study. This achievement further expands the available genomic resources for human medical genetics research and will contribute to the provision of precision medical services to diverse populations. This study focused on several reported genes associated with the progression of dental caries that have variants between the Han1 and BKY populations, including *PRB4*, which encodes a proline-rich alkaline salivary protein (Figure 2c). The deletion of the amino acid sequence encoded by *PRB4* in the BKY genome is noteworthy, previous studies have reported that the salivary peptides derived from the breakdown of this protein are larger in caries-susceptible individuals compared to those found in caries-free individuals’ saliva [60]. Consistent with previous study, our results also showed that the amino acid sequence encoded by *PRB4* is shorter than that of Han1. Additionally, the amino acid sequences encoded by *PRB1* and *PRB3* are also shorter in length due to deletion compared to Han1. Defensins are an important component of the oral defense system, as they possess both direct antimicrobial effects and the ability to modulate host immunity [62]. The functional polymorphisms of *DEFB1* gene have been identified as potential markers for dental caries [61]. It was found that a single nucleotide mutation occurred in the CDS of *DEFB134* gene, resulting in a substitution of a nonpolar amino acid with a polar amino acid in the encoded protein. Between BKY and Han1, a deletion of an α helix segment has been identified in DEFB134 (Figure 2d). The structure of DEFB126 has also been found to differ between BKY and Han1, with a reversal of hydrophilicity and hydrophobicity observed at its mutation sites (Supplementary Fig. S12). These variations may affect the antimicrobial activity of the peptide, thus leading to a reduction in the incidence of dental caries. Furthermore, compared to Han1, ZNF160 exhibits structural differences, and hydrophilicity enhancement is observed at the mutation sites (Supplementary Fig. S12).

The occurrence of dental caries is influenced by multiple factors, with estimates suggesting that genetic factors account for more than 50% of the variation [63,64]. In this study, multiple sequence variations detected between BKY and Han1 genomes were found to be located in the coding regions of genes related to dental caries, which further led to changes in the sequence and structure of the corresponding proteins, and ultimately affected the function of the corresponding proteins. This is thought to influence the differences in caries susceptibility between the BKY and Han populations and ultimately lead to the observed differences in caries incidence between the two populations. This further provides evidence for the genetic basis of dental caries occurrence.

Increased phospholipids such as phosphatidylcholine, sphingolipids, and lysophosphatidylcholine were found in the caries group of the BKY, which is consistent with previous studies on the association between phospholipids and the mechanism of dental caries [65,66]. Phospholipids are constituents of dental biofilms affecting the adsorption of various proteins and molecules on the surface of teeth or biofilms and participating in the growth and metabolism of oral microbes [67]. Additionally, the presence of phospholipids is considered to have a significant effect on the toughness and thickness of biofilms [65]. With the preservation of biofilm thickness and toughness, acid-resistant microbes associated with dental caries, such as *Streptococcus mutans* and *Lactobacillus acidophilus*, can settle and grow on the tooth surface, metabolizing carbohydrates into acidic compounds. However, an increase in phospholipid levels may inhibit the diffusion of acidic compounds, leading to demineralization of the tooth surface and formation of initial lesions [66]. These findings indicate the close relationship between oral microbes and phospholipids as well as their synergistic effects in the onset of dental caries.

Increased levels of Gln-Glu, Pyroglu-Ile-Arg, concanamycin b, and benzamide were found in the caries-free group of the BKY population; these substances function differently in various physiological and metabolic pathways. Some metabolites may be related to oral microbes and dental caries, while others may involve overall human metabolism and immune status. Gln-Glu is a complex of glutamic acid and glutamine and is the major source of ammonia production at high concentrations, helping to neutralize acidic environments, stabilize pH, and prevent acid-induced enamel demineralization on tooth surfaces [68]. Pyroglu-Ile-Arg is a small peptide rich in arginine, and cleavage of these peptides increases the level of free arginine in saliva, promoting ammonia production for acid neutralization [69] and reducing susceptibility to dental caries [24]. Furthermore, arginine can activate the microbial arginine deiminase system, leading to resistance against tooth surface demineralization. Toothpaste containing arginine has been suggested to possess anticaries properties [70]. Concanamycin b is mainly produced by Streptomyces and is a macrolide antibiotic with antifungal, antiviral, and immunomodulatory activities [71], but the mechanism underlying its inhibition of caries-causing microbes in the oral cavity has not yet been reported. Benzamide is an aromatic compound that might impact the metabolism and growth of oral microbes. Its specific mechanisms and relationship with dental caries require further research. However, it has been reported that benzamide-like substances produced by *Streptomyces* can inhibit *Candida albicans*, *Escherichia coli*, and other microbes [72] and that benzoates inhibit the growth of caries-causing oral microbes in an acidic environment in a similar manner to fluoride [73].

In terms of microbial communities, the predominant genera enriched in the dental caries group of the BKY population were *Prevotella*, *Veillonella*, and *Actinomyces*. These microbes are reportedly associated with the onset and progression of dental caries [6–8]. Moreover, caries-causing microbes such as *Actinomyces viscosus* (*P* value < 0.05) and *Streptococcus mutans* (*P* value > 0.05) showed higher abundances in the dental caries group than in the caries-free group. Within the caries-free BKY group, *Neisseria* and *Capnocytophaga* exhibited higher abundance (Figure 4 and Supplementary Table S8). *Neisseria* species typically inhabit human oral and nasopharyngeal cavities but are generally not pathogenic. Some members of this genus can metabolize lactate in the oral cavity to reduce local acidification of the microenvironment [74]. *Capnocytophaga* species were found to be more abundant in the caries-free group in previous studies [75]. Additionally, they were shown to decrease in abundance with disease progression in studies by Gross and Kahharova [5,76]. These studies suggest that *Capnocytophaga* can exist in the core oral microbiota of a healthy population, which our study provides new molecular evidence for.

Dysfunction of microbial functions can also serve as an important reflection of disease. The enrichment of CAMP resistance genes suggests that the oral microbiota of the BKY population may have experienced long-term exposure to CAMPs. Modifying the lipopolysaccharide structure is an important mechanism by which microbes resist CAMPs, which can reduce their binding and killing activity [77]. The CAMP resistance gene cluster is primarily derived from dominant microbial genera in the oral cavity, such as Streptococcus, Haemophilus, and Neisseria. However, common caries-causing bacteria, such as Streptococcus mutans [78], Lactobacillus acidophilus [79], and Actinomyces viscosus [80], almost completely lack this gene cluster. This provides a genetic foundation for inhibiting caries-causing microbes with antimicrobial peptides and promoting beneficial Streptococcus (Streptococcus salivarius or Streptococcus oralis) to become the dominant microbial community. Additionally, beneficial Streptococcus species have been found to inhibit the growth of caries-causing microbes in several studies [80,81]. In addition, these dominant microbial genera, such as Streptococcus, Haemophilus, and Neisseria, can maintain oral microbial homeostasis, thereby maintaining oral health and avoiding dental caries caused by an imbalance in the resident microbial community [82].

In our combined metagenome and metabolomic analyses, we found that the differential metabolites negatively associated with caries-causing microbes were mostly small-molecule peptides, such as Ac-Pro-Gly-Pro, Arg-Pro-Pro, Gln-Glu, Lys-Pro, and Pyroglu-Ile-Arg. These small peptides rich in proline may have been generated through the degradation of basic proline-rich proteins encoding by PRB gene family or proline-rich antimicrobial peptides. It is speculated that these active substances could inhibit the growth of caries-causing microbes by neutralizing acidic environmental conditions or acting directly on those microbes [68,69].

**Figure 5.**
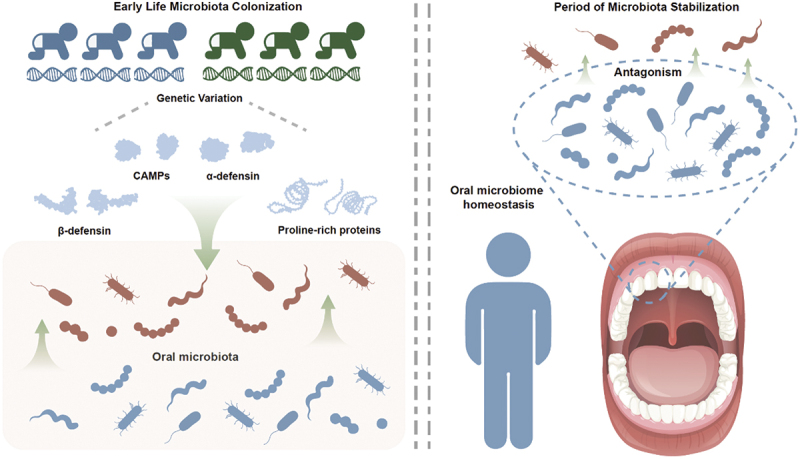
Diagram illustrating the establishment of oral microbiota homeostasis in early life through the promotion of antimicrobial metabolites. Caries-causing microbes are shown in red. The core microbiota of a healthy oral cavity is shown in blue. The caries-associated genetic variation between the genomes of the BKY and Han Chinese populations may involve the *PRB4* and *DEFB134*. The diagram on the right reflects the inhibition of caries-causing microbes by microbiota homeostasis in the oral cavity. The graphic was made with assets from Freepik.com.

There may be multiple causal factors involved in the onset and progression of dental caries [2,3], and these factors must be considered for dental caries prevention and control [83]. Many cationic antimicrobial peptides are present in saliva, such as α-defensins (HNP1–4), β-defensins (HBD1–3), and LL-37, which can play a role in oral innate defense [20]. Based on genomic comparisons, we hypothesize that there may be highly effective caries-suppressing genotypes present in the BKY population, such as *DEFB134* and *PRB4*. These genes produce many cationic antimicrobial peptide metabolites during infancy and early childhood, which can significantly reduce colonization by caries-causing microbes (such as *Streptococcus mutans* and *Lactobacillus acidophilus*) in the early stages of oral ecosystem formation [24,84,85]. Additionally, as a result of long-term exposure to antimicrobial peptides, the oral microbiota in the BKY population has predominantly retained microbial taxa (such as *Streptococcus* and *Neisseria*) with gene clusters capable of resisting antimicrobial peptides. Moreover, microbial communities that are mainly composed of these microbes can competitively inhibit caries-causing microbes over an extended period [18], promoting long-term oral health despite increased sugar intake and exposure to a more complex microbial environment (Figure 5). Our study has revealed new clues and evidence regarding the specific regulatory mechanism underlying the low prevalence of dental caries in the BKY population and provides a basis for the genetic diagnosis and control of dental caries.

We acknowledge the limitations inherent in exploratory research on the oral microbiome and metabolome within BKY populations. The small sample size used in this study, as a result of stringent inclusion criteria, may not fully represent the oral microbiome across all BKY populations. Due to the small sample size, non-corrected *P* values and species with > 0.1% abundance were used when multiple comparisons were performed. We obtained compelling preliminary evidence from metagenomic and metabolomic datasets. Specifically, we found that the oral microbiota diversity in the caries-free group was higher than that in the caries group, which is consistent with previously reported studies [86,87]. However, further longitudinal, properly controlled studies with larger cohorts and independent confirmation cohorts are needed to determine valid disease biomarkers for and clarify the role of oral microbes and metabolites in the development of caries.

## Supplementary Material

Supplemental Material

## Data Availability

The raw data of genome and metabolome sequencing are available from the National Genomics Data Center [88] under project number PRJCA017955, PRJCA019103 and PRJCA019104.
