# Supplementary material for: Multiomics analysis reveals the genetic and metabolic characteristics associated with the low prevalence of dental caries
Source: J Oral Microbiol. 2023 Nov 2;15(1):2277271. doi: 10.1080/20002297.2023.2277271 (PMC10623897; doi:10.1080/20002297.2023.2277271)
Supplement: Supplemental Material [file ZJOM_A_2277271_SM6590.zip › Supplementary files/Supplementary_Figure_v3.pdf]

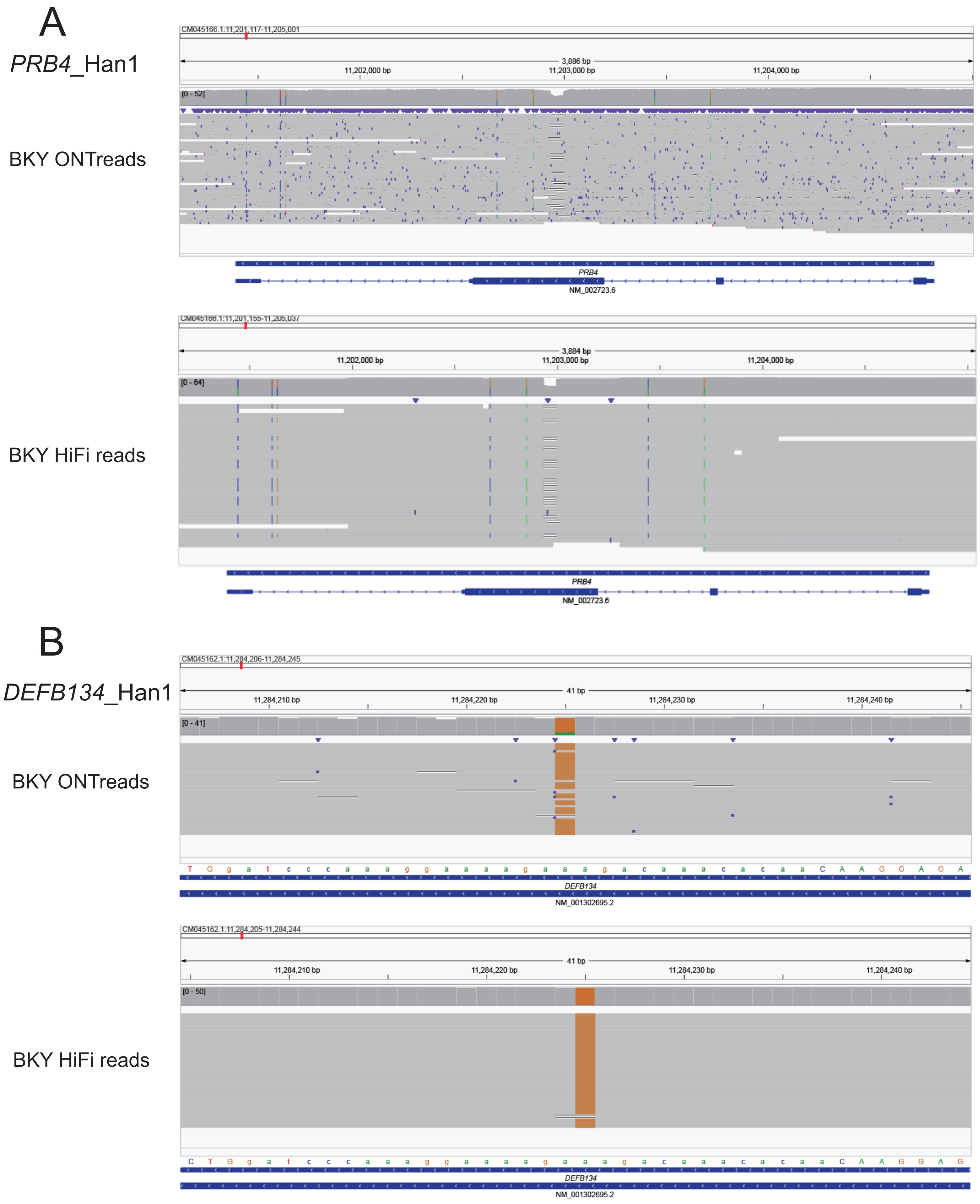

Supplementary Fig. 1. Validation of variation in *PRB4* and *DEFB134* between Han1 and BKY using PacBio HiFi and ONT reads. A. Coverage of PacBio HiFi and ONT reads in the region where *PRB4* is located. **B.** Coverage of PacBio HiFi and ONT reads in the region where *DEFB134* is located.

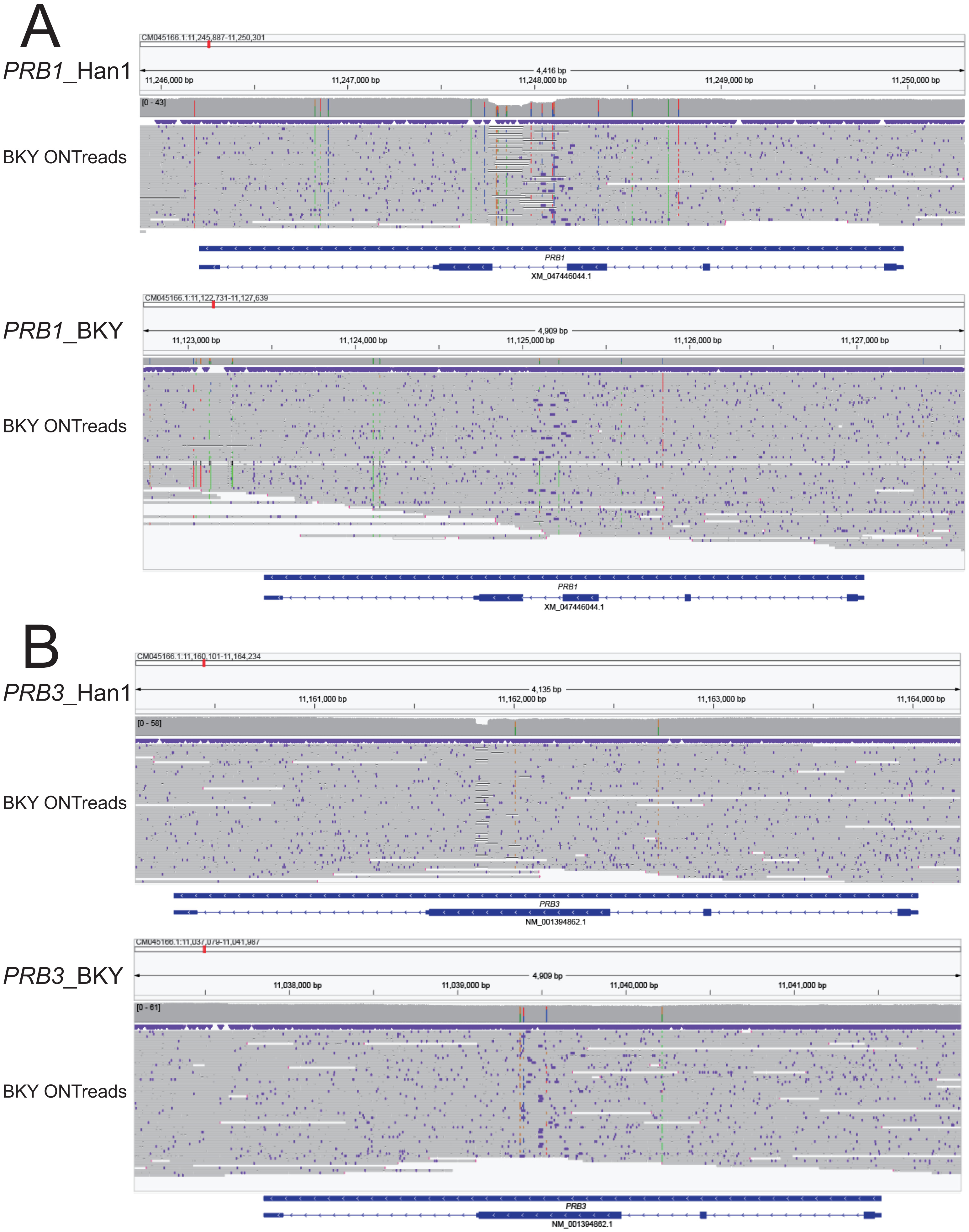

Supplementary Fig. 2. Validation of variation in *PRB1* and *PRB3* between Han1 and BKY using ONT reads. A. Coverage of ONT reads in the region where *PRB1* is located. **B.** Coverage of ONT reads in the region where *PRB3* is located.

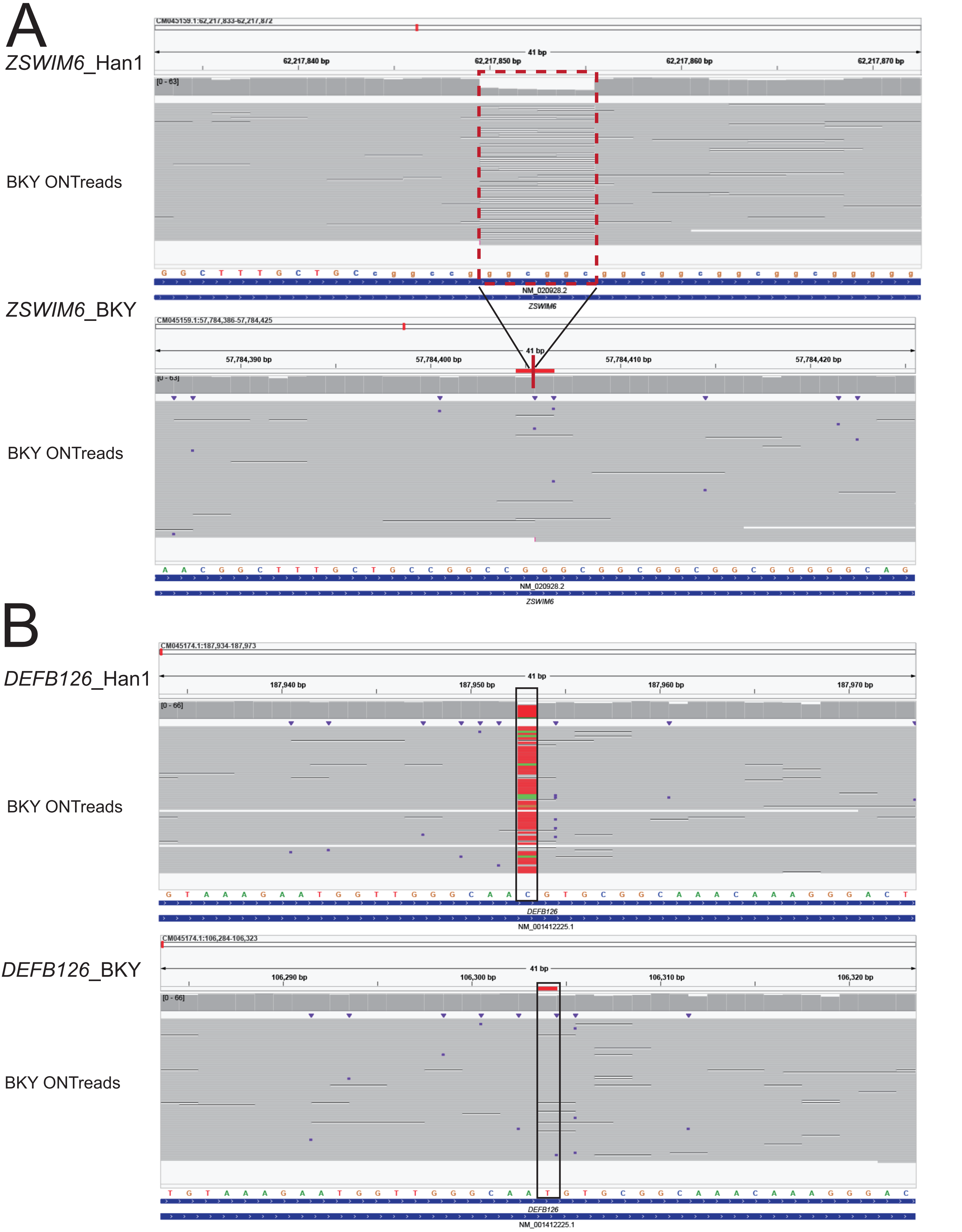

Supplementary Fig. 3. Validation of variation in *ZSWIM6* and *DEFB126* between Han1 and BKY using ONT reads. A. Coverage of ONT reads in the region where *ZSWIM6* is located. **B.** Coverage of ONT reads in the region where *DEFB126* is located.

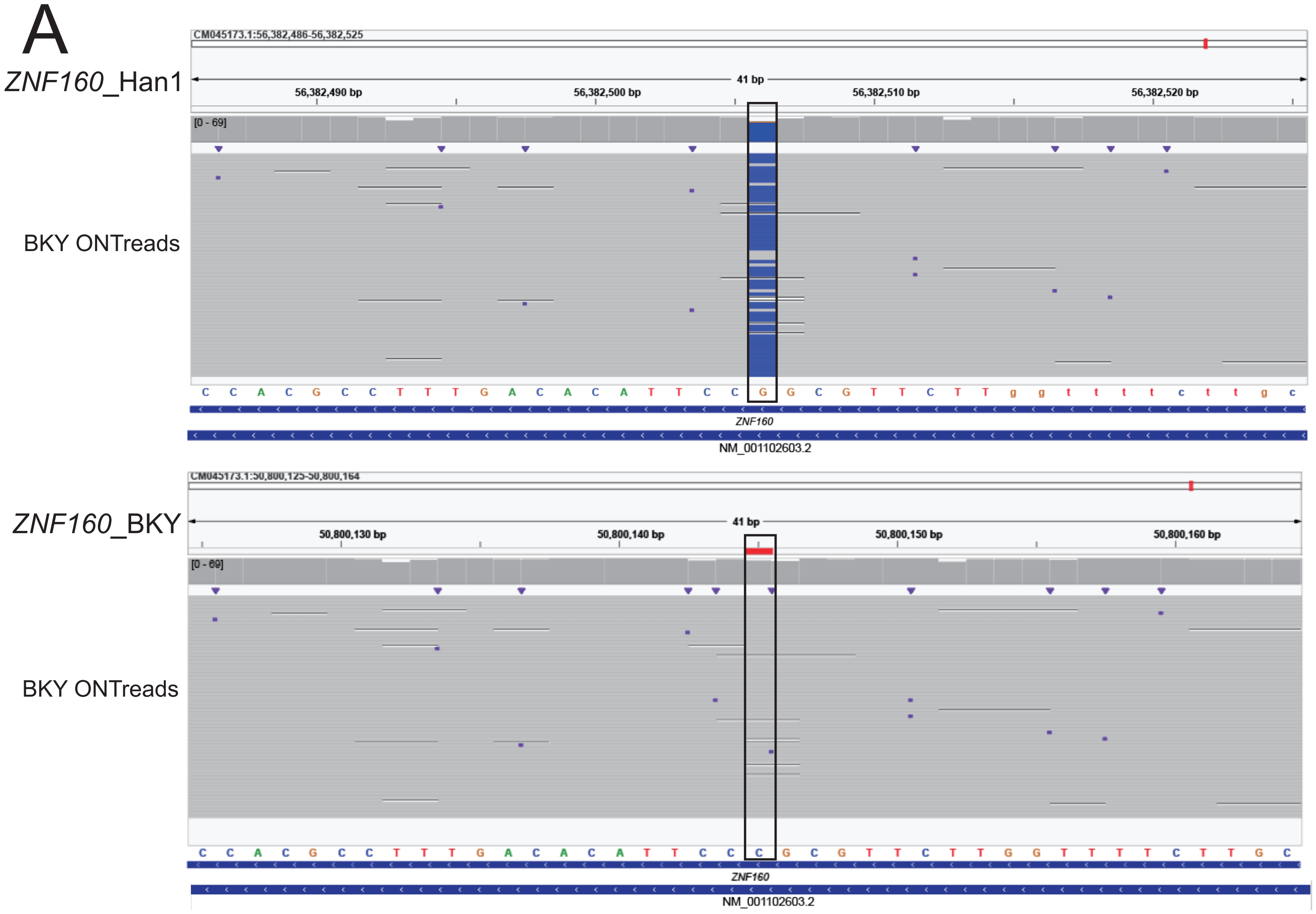

Supplementary Fig. 4. Validation of variation in *ZNF160* between Han1 and BKY using ONT reads. A. Coverage of ONT reads in the region where *ZNF160* is located.

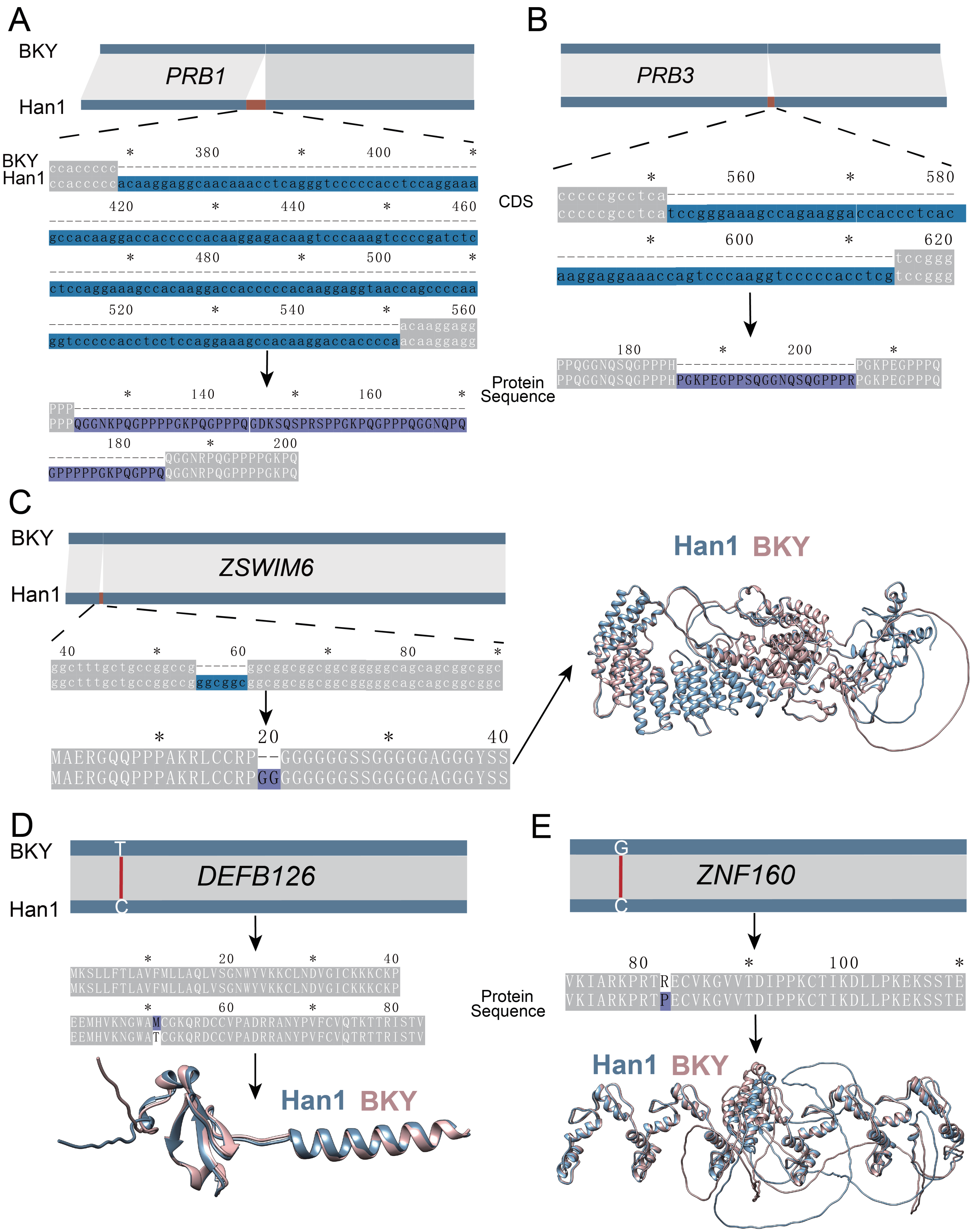

Supplementary Fig. 5. Variation of caries related genes between BKY and-Han1 genomes. C. ZSWIM6, the calculated RMSD was 0.316 Å, and TM-score was 0.952. **D.** DEFB126, the calculated RMSD was 0.855 Å, and TM-score was 0.841. **E.** ZNF160, the calculated RMSD was 0.832 Å, and TM-score was 0.837.

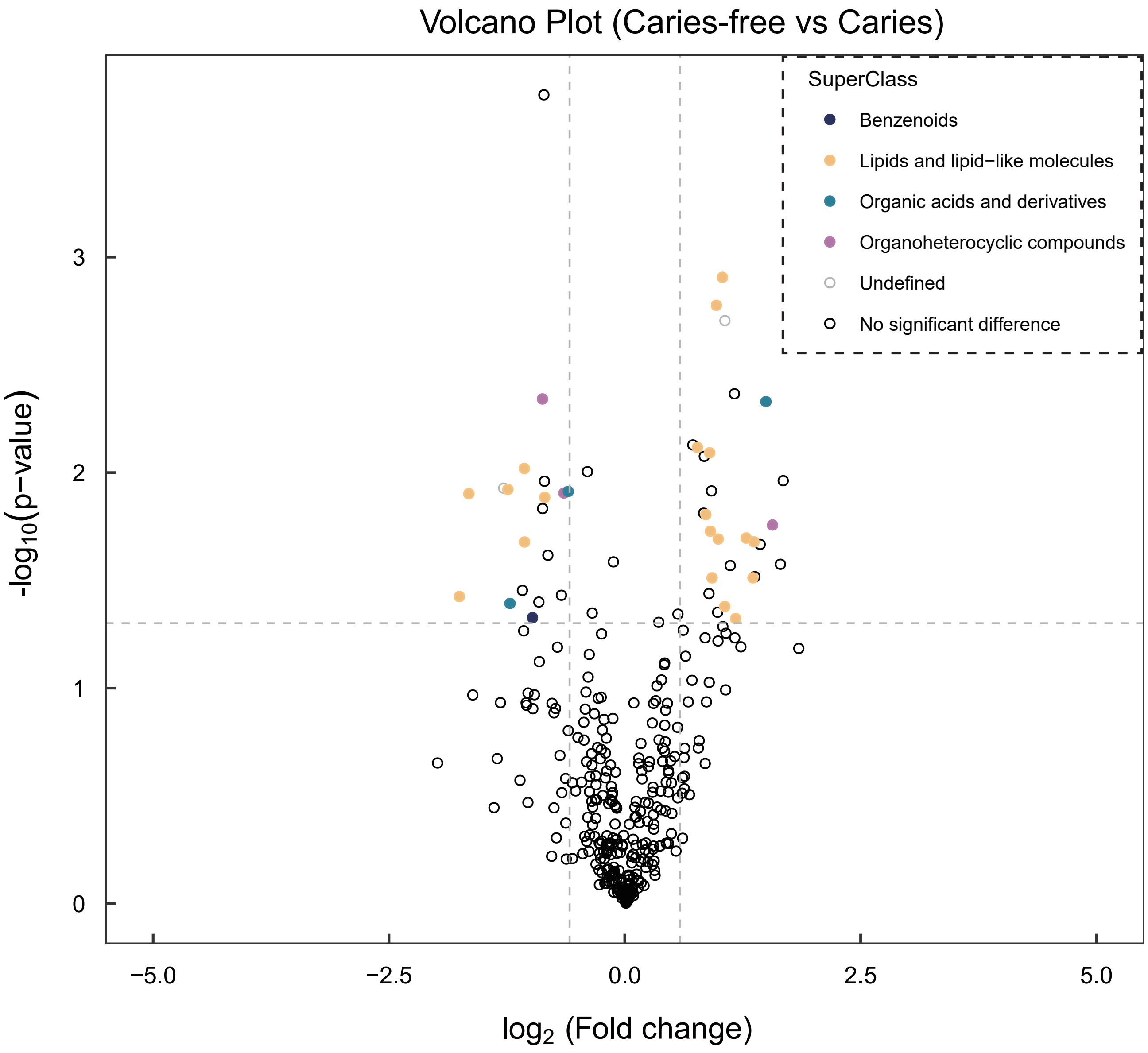

Supplementary Fig. 6. The volcano plot of saliva sample comparison (caries/caries-free). Significant differential metabolites (negative mode): FC > 1.5 or FC < 0.67; *P* value < 0.05.

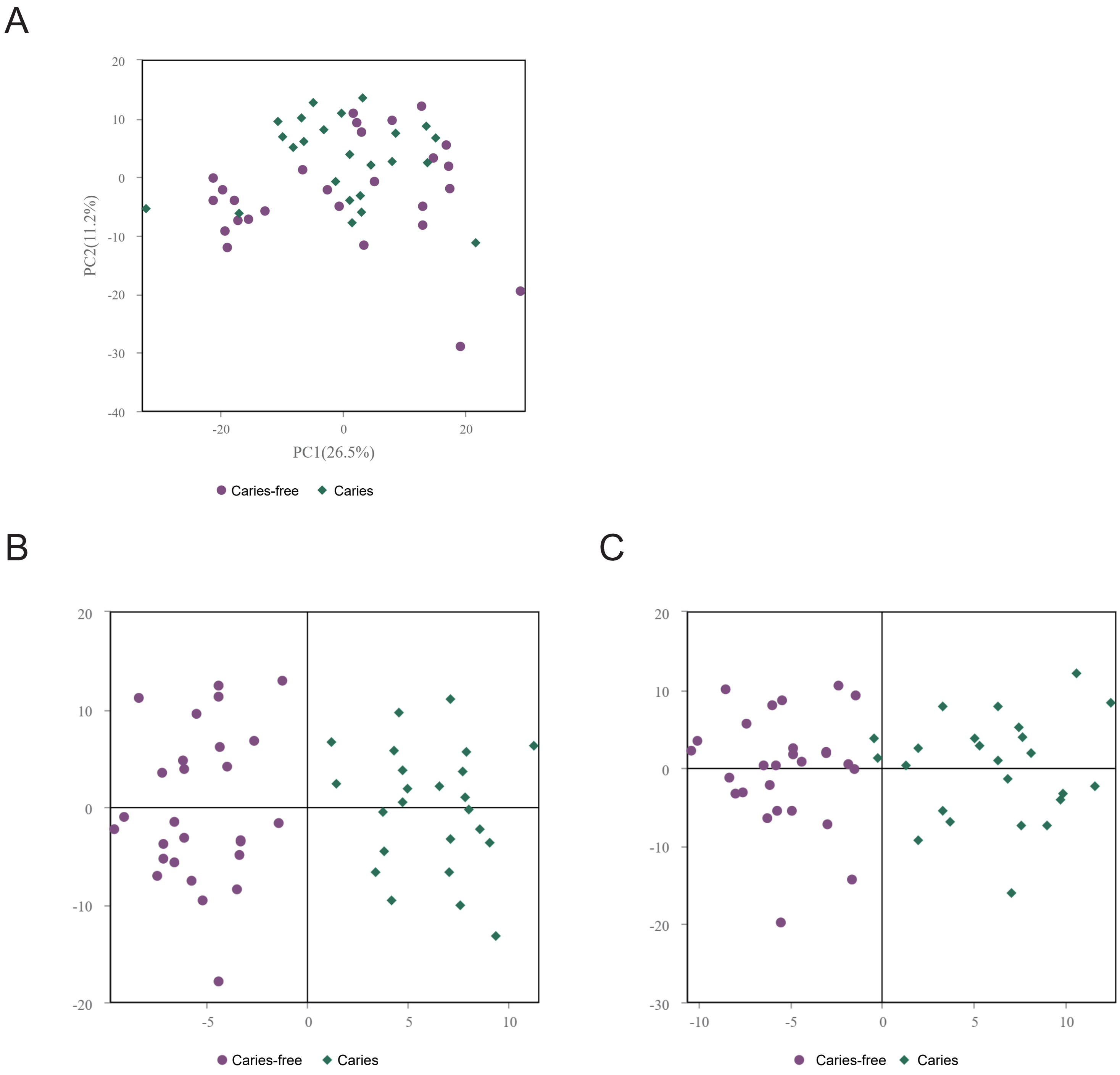

Supplementary Fig. 7. A. PCA from the caries and caries-free groups (negative mode). **B.** OPLS-DA from the caries and caries-free groups (positive mode). **C.** OPLS-DA from the caries and caries-free groups (negative mode).

A

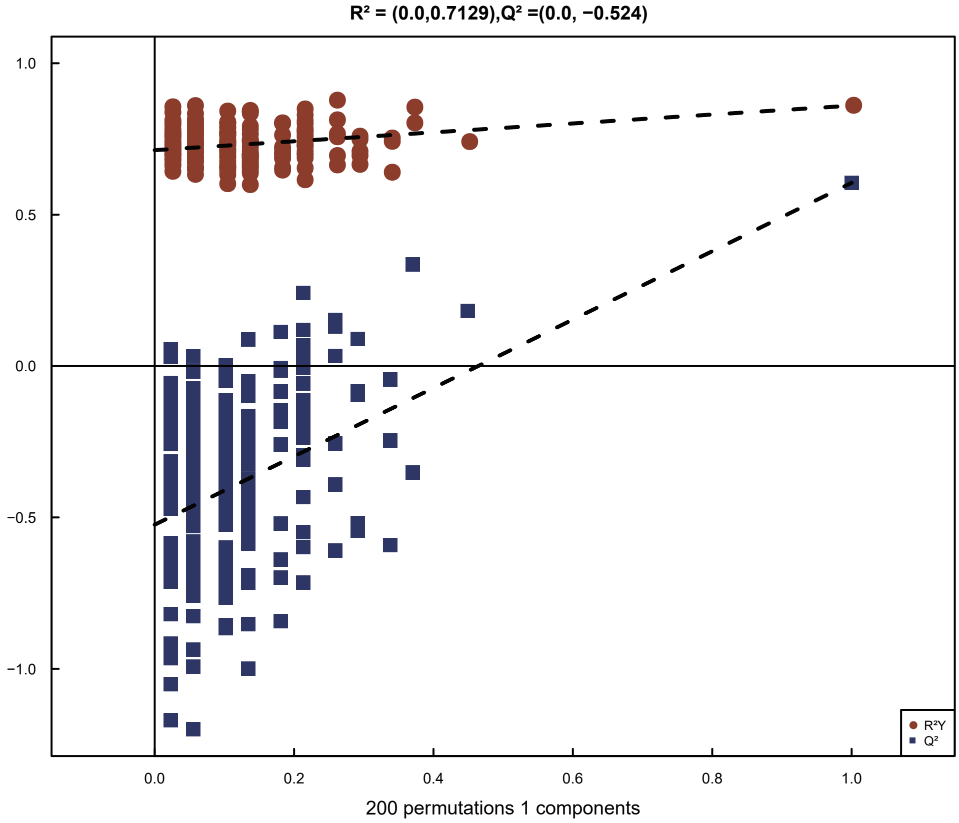

B

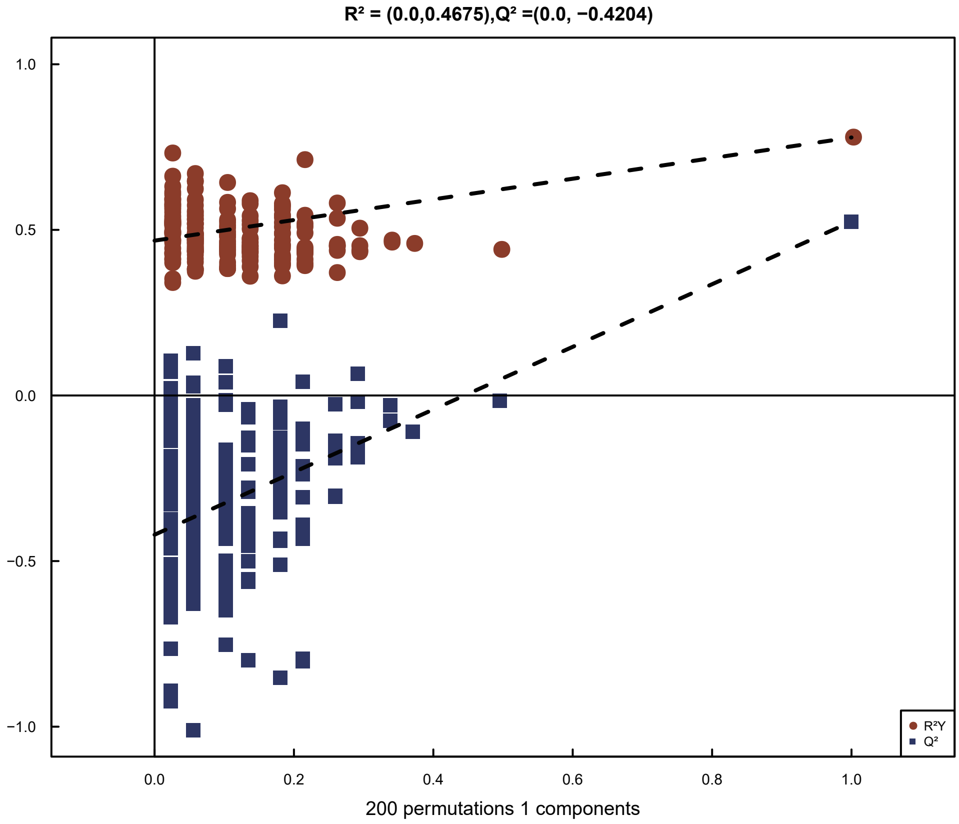

Supplementary Fig. 8. A. The Permutation test of OPLS-DA (positive mode). **B.** The Permutation test of OPLS-DA (negative mode).

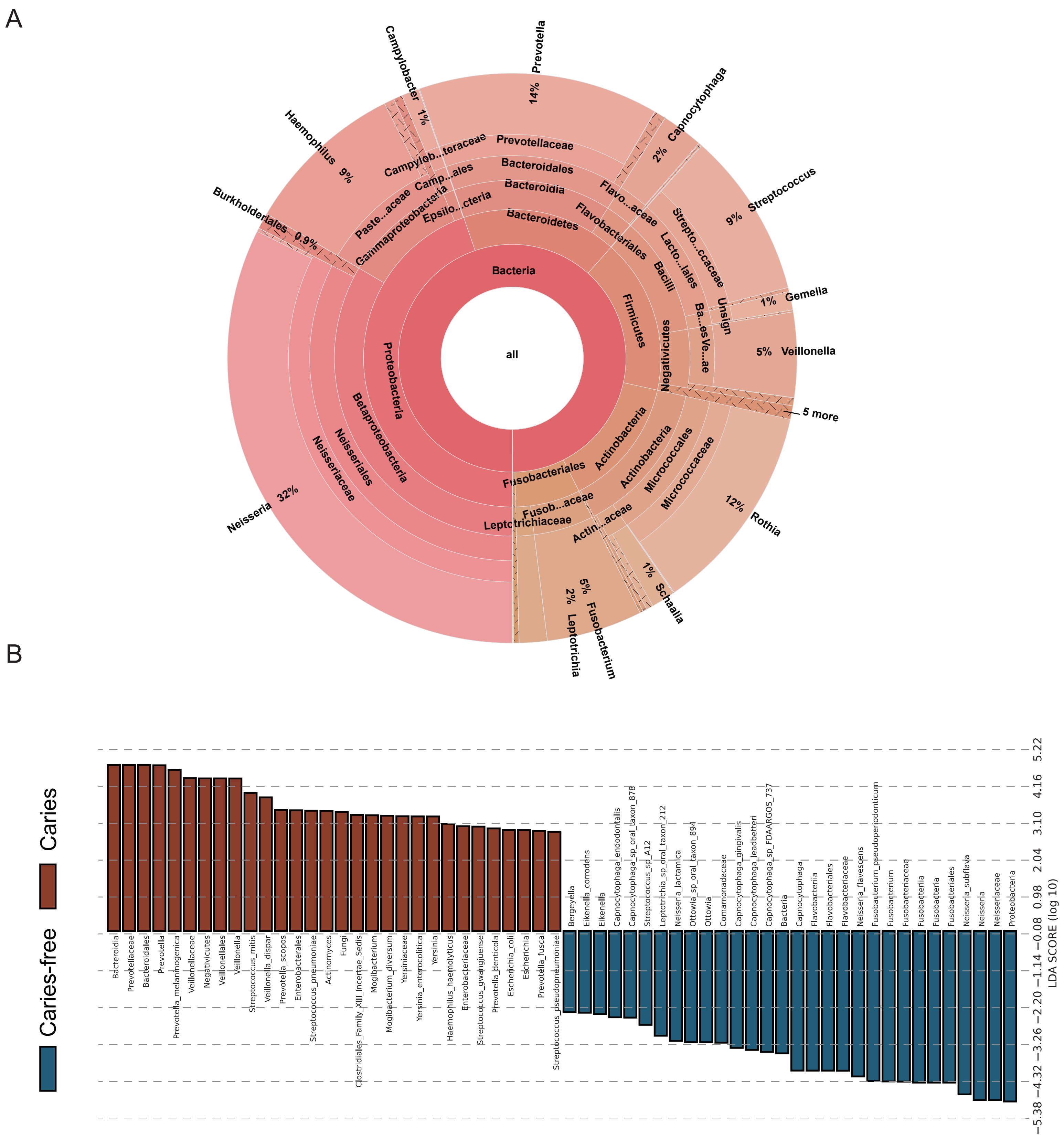

Supplementary Fig. 9. A. The Krona plots of species annotation results at different taxonomic levels. **B.** LDA score plot generated from LEfSe analysis.

A

B

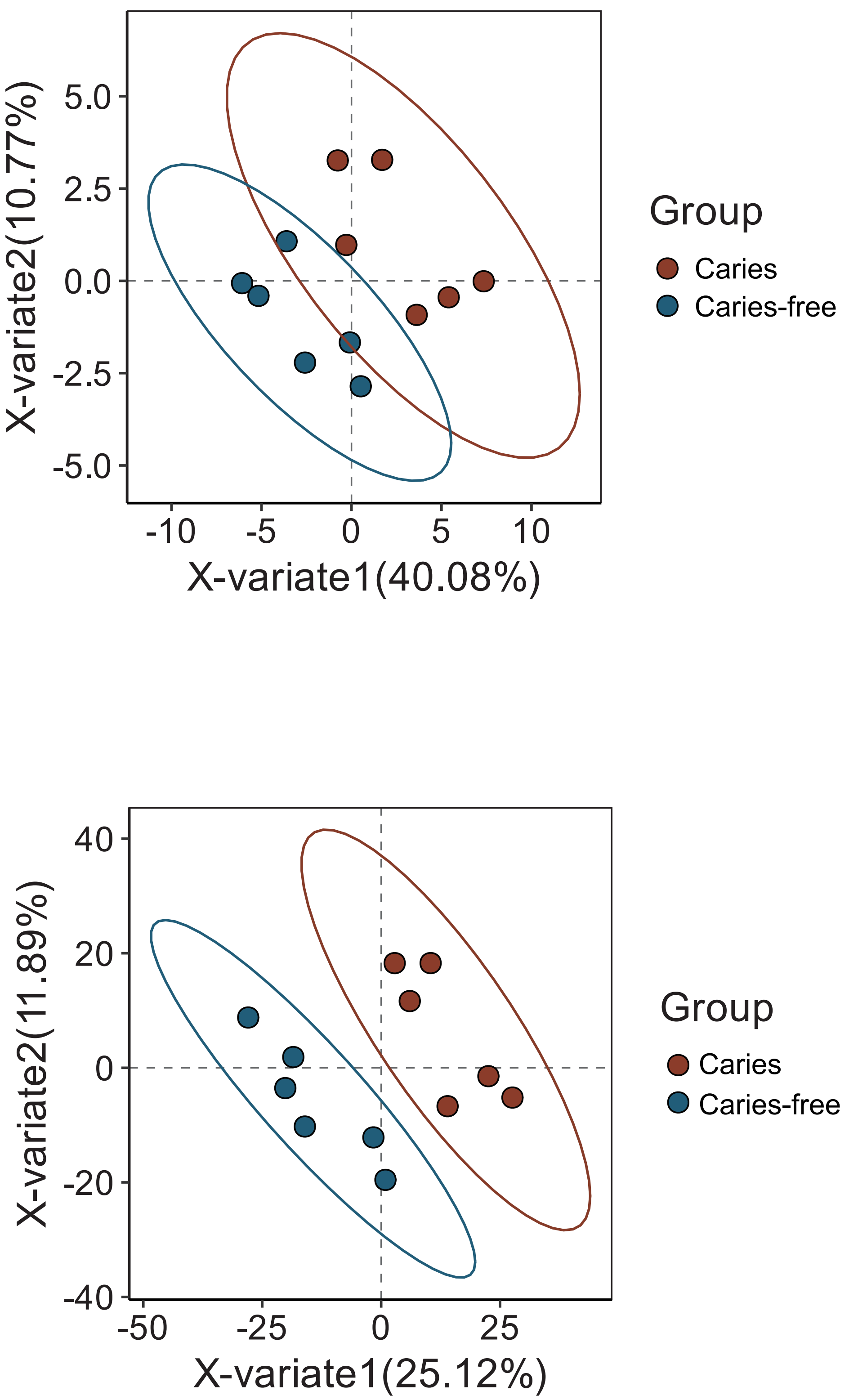

Supplementary Fig. 10. A. PLS-DA clustering for oral saliva samples from 12 persons at the phylum level. **B.** PLS-DA clustering for oral saliva samples from 12 persons at the genus level.

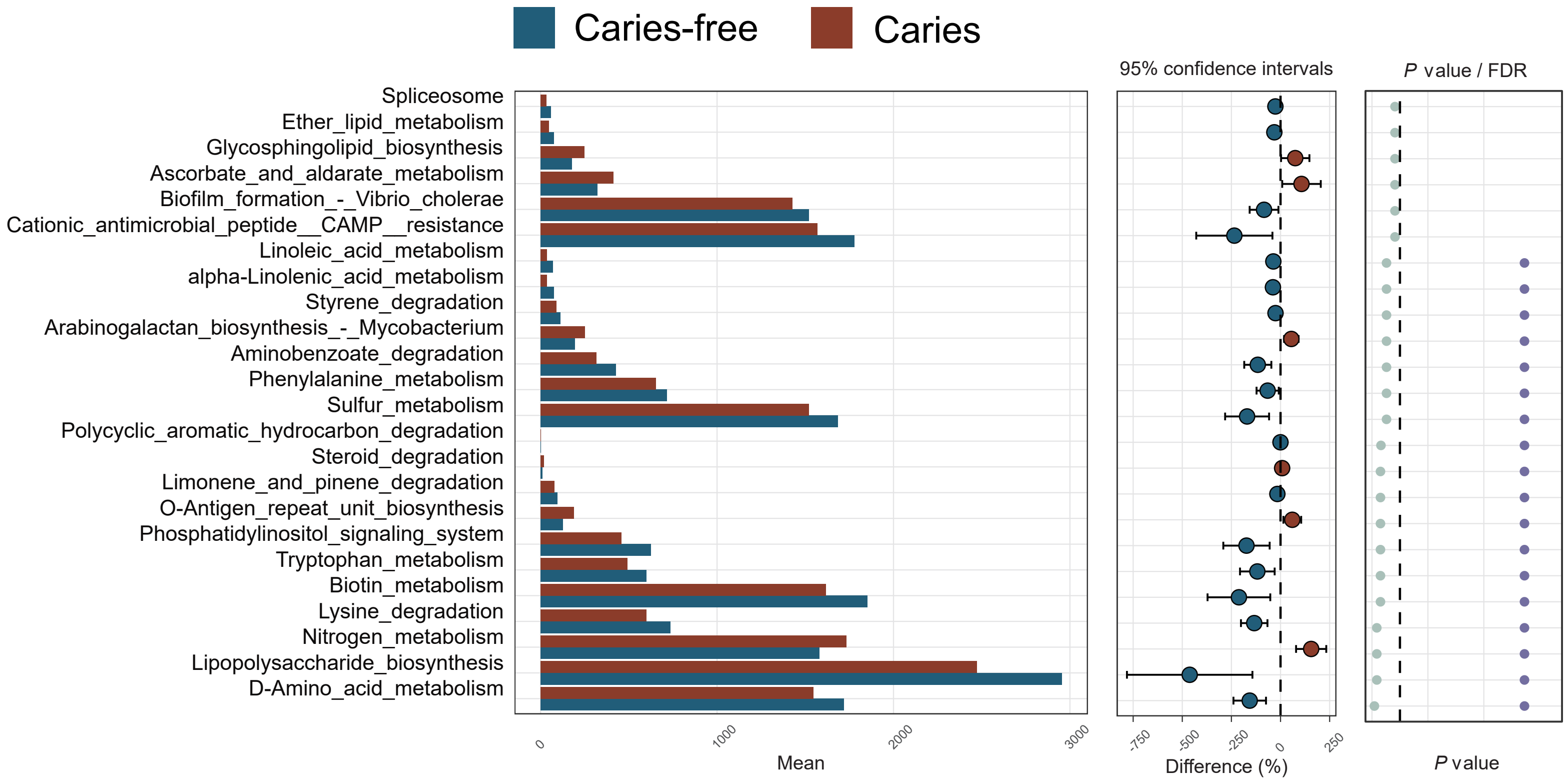

Supplementary Fig. 11. Differential microbial function analysis between groups using STAMP.

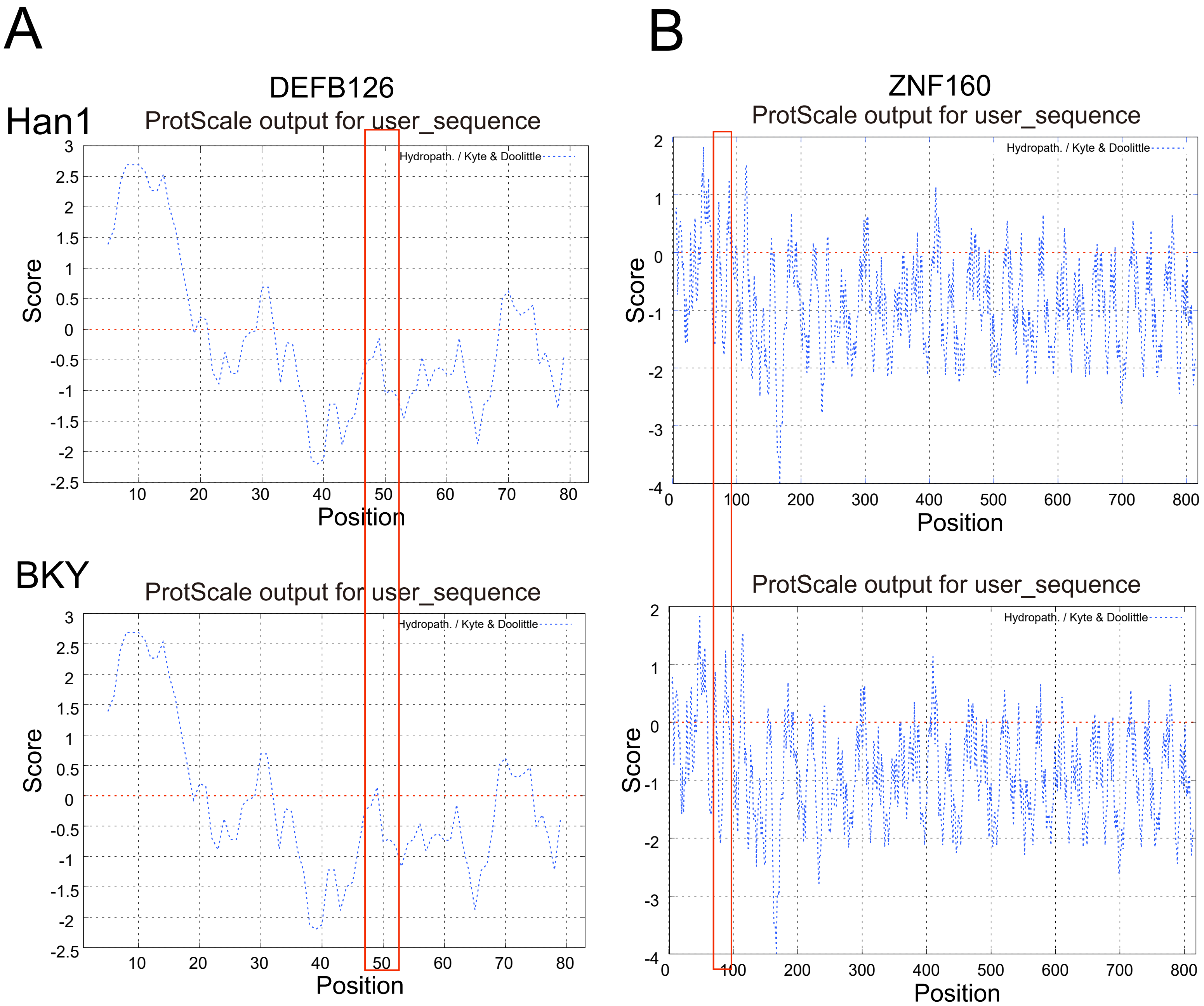

Supplementary Fig. 12. Comparison of hydrophilicity and hydrophobicity of DEFB126 and ZNF160 between BKY and Han1. A. Comparison of hydrophilicity and hydrophobicity of DEFB126 between BKY and Han1. Positive values are hydrophobic and negative values are hydrophilic. **B.** Comparison of hydrophilicity and hydrophobicity of ZNF160 between BKY and Han1.
